# Supplementary material for: Severity and mortality prediction models to triage Indian COVID-19 patients
Source: PLOS Digit Health. 2022 Mar 9;1(3):e0000020. doi: 10.1371/journal.pdig.0000020 (PMC9931227; doi:10.1371/journal.pdig.0000020)
Supplement: S1 Text — Fig A: The points are colored according to the clustering labels (learned from data), and the point shape represents the severity status of the patients. Table A: Comparison of features in deceased and alive patients in the mortality prediction model. Table B: Comparison of features in severe and non-severe patients in the severity prediction model. Fig B: Distribution of features with high predictive power. Table C: Evaluation of reduced mortality model. Table D: Evaluation of reduced mortality model (DOCX) [file pdig.0000020.s001.docx]

# **Supplementary Material**

# **1. Clustering to visualize clinical bias**

For maintaining uniformity and removing skewness from the distributions of the features, we transformed the data using PowerTransformer of the sklearn.preprocessing module, which implements the Yeo-Johnson transformation. [[1]](https://paperpile.com/c/8FRoID/Cnro)

The clustering was done using the KPrototypes [2] algorithm implemented in the kmodes package using the number of clusters as 2 since we had two classes (severe and non-severe). The umap [3] and plotly.express [4] packages were used to reduce the dimensionality of the data and make the plots, respectively.
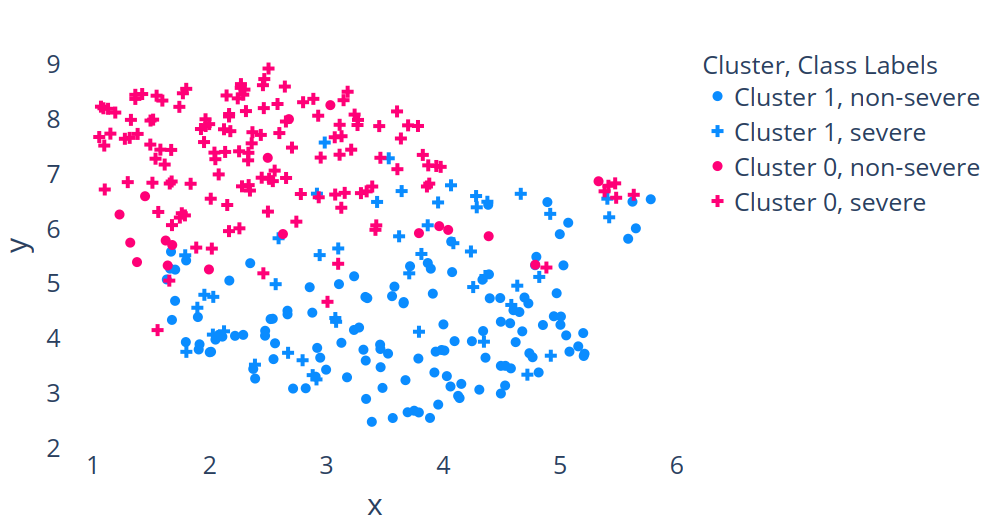


**Fig A:** The points are coloured according to the clustering labels (learned from data), and the point shape represents the severity status of the patients.

# **2. Description of gross clinical parameters collected.**

| Features | Alive Patients | Deceased Patients |
| --- | --- | --- |
|  | Median (Interquartile Range) | Median (Interquartile Range) |
| AST / SGOT (U/L) | 41.4 (36.76, 65.1) | 55.1 (36.76, 86.10) |
| Age (Years) | 49 (36, 60) | 70 (62, 79) |
| Alkaline phosphatase (U/L) | 92.4 (75.15, 119.8) | 109.77 (83.53, 134.93) |
| Direct Bilirubin (mg/dL) | 0.28 (0.2, 0.39) | 0.3 (0.21, 0.41) |
| Lymphocyte (%) | 21 (13, 29) | 9 (5, 15) |
| N/L Ratio | 3.3 (2.14, 6.08) | 9.44 (5.06, 17.9) |
| Platelet Count (10^5^cells/µL) | 1.62 (1.16, 2.38) | 1.85 (1.35, 2.77) |
| RBC Count (cells/mcL) | 4.41 (4, 4.82) | 4.42 (3.98, 4.85) |
| SGPT (U/L) | 39.6 (24.5, 70) | 41.38 (21.95, 66.07) |
| Serum Potassium (mmol/L) | 4.5 (4.03, 4.91) | 4.5 (4.03, 5.05) |
| Total Bilirubin (mg/dL) | 0.65 (0.49, 0.84) | 0.78 (0.54, 1.03) |
| Urea (mg/dL) | 25.1 (19.8, 32.6) | 48.05 (31.18, 80.88) |
| WBC Count (cells/µL) | 6800 (5300, 8900) | 11300 (7200, 17950) |
| Basophil (%) | 0 (0, 0) | 0 (0, 0) |
| Creatinine (mg/dL) | 1 (0.87, 1.16) | 1.33 (1.0, 1.68) |
| Eosinophils (%) | 2 (1, 3) | 1 (1, 2) |
| Hematocrit (%) | 39.5 (35.5, 42.5) | 38.1 (34, 42.3) |
| Hemoglobin (g/dL) | 12.5 (11.3, 13.8) | 12.25 (10.9, 13.98) |
| Indirect Bilirubin (mg/dL) | 0.35 (0.24, 0.48) | 0.42 (0.28, 0.67) |
| Mean Corpuscular Hemoglobin (pg) | 28.7 (26.9, 30.7) | 28.55 (25.73, 30.98) |
| Mean Corpuscular Volume (fL) | 90.4 (85.8, 96.2) | 88.25 (81.95, 93.33) |
| Monocytes (%) | 6 (4, 10) | 4 (2, 6) |
| Neutrophils (%) | 69.9 (60, 79) | 86 (77.25, 90) |
| Outcome (0: Deceased  1: Alive) | 0 (0, 0) | 1 (1, 1) |
| Serum Sodium (mmol/L) | 140 (136.1, 143.2) | 137.75 (133.85, 141.73) |
| Gender (0: M  1: F) | 0 (0, 1) | 0 (0, 1) |

**Table A:** Comparison of features in deceased and alive patients in the mortality prediction model

| Features (Units) | Severe Patients | Non-Severe Patients |
| --- | --- | --- |
|  | Median (Interquartile Range) | Median (Interquartile Range) |
| AST / SGOT (U/L) | 37.1 (26.65, 53.98) | 51.4 (36.53, 78.08) |
| Age (Years) | 50 (41, 63.25) | 65 (55, 75) |
| Alkaline phosphatase (U/L) | 90.64 (71.35, 117.4) | 101.2 (78.48, 126) |
| Direct Bilirubin (mg/dL) | 0.26 (0.2, 0.38) | 0.3 (0.21, 0.39) |
| Lymphocyte (%) | 23 (15, 31) | 12 (7, 17.95) |
| N/L Ratio | 2.89 (1.87, 4.89) | 7 (4, 12.86) |
| Platelet Count (10^5^cells/µL) | 1.52 (1.06, 1.99) | 2.06 (1.44, 3.15) |
| RBC Count (cells/mcL) | 4.41 (3.96, 4.81) | 4.33 (3.94, 4.8) |
| SGPT (U/L) | 37.65 (21.2, 61.53) | 43.67 (24.21, 75) |
| Serum Potassium (mmol/L) | 4.47 (4.12, 4.9) | 4.4 (3.99, 4.89) |
| Total Bilirubin (mg/dL) | 0.58 (0.44, 0.79) | 0.68 (0.52, 0.94) |
| Urea (mg/dL) | 25.45 (21.18, 31.08) | 40.9 (28.86, 59.87) |
| WBC Count (cells/µL) | 6200 (5100, 7725) | 10900 (7010, 15750) |
| Basophil (%) | 0 (0, 0) | 0 (0, 0) |
| Creatinine (mg/dL) | 1.045 (0.91, 1.26) | 1.17 (0.95, 1.42) |
| Eosinophils (%) | 2 (1, 3) | 1 (1, 2) |
| Hematocrit (%) | 38.7 (34.98, 42.53) | 38.5 (34.25, 41.8) |
| Hemoglobin (g/dL) | 12.4 (11.2, 13.5) | 12.1 (10.85, 13.5) |
| Indirect Bilirubin (mg/dL) | 0.305 (0.21, 0.41) | 0.38 (0.27, 0.53) |
| Mean Corpuscular Hemoglobin (pg) | 28.5 (26.7, 30.5) | 28.1 (26.05, 30.25) |
| Mean Corpuscular Volume (fL) | 88.7 (84.65, 93.73) | 88.9 (82.55, 93.65) |
| Monocytes (%) | 7 (4, 10) | 3 (2, 6) |
| Neutrophils (%) | 66 (58, 76) | 83 (73.35, 89) |
| Outcome (0: Deceased  1: Alive) | 0 (0, 0) | 1 (1, 1) |
| Serum Sodium (mmol/L) | 140.65 (137.2, 143.6) | 137.5 (133.5, 141.3) |
| Gender (0: M  1: F) | 0 (0, 1) | 0 (0, 1) |
| D-D dimer (ng/mL) | 354 (241.5, 454.3) | 780 (483, 1763.55) |
| Ferritin (ng/mL) | 244 (109.93, 480.68) | 544 (260.6, 1196.25) |
| High sensitivity C-reactive protein (mg/L) | 24.75 (6.21, 47.8) | 62 (25.98, 94.63) |

**Table B:** Comparison of features in severe and non-severe patients in the severity prediction model

**3. Description of clinical parameters used in reduced mortality prediction models**

This is the supplementary document containing information about the distributions of the features that were used and were found to be of importance in the mortality and severity models. These features are:

1. D-D Dimer
2. Ferritin
3. High Sensitivity C - Reactive Protein
4. Age
5. Urea
6. Alkaline Phosphatase
7. Creatinine
8. Indirect Bilirubin
9. Neutrophil
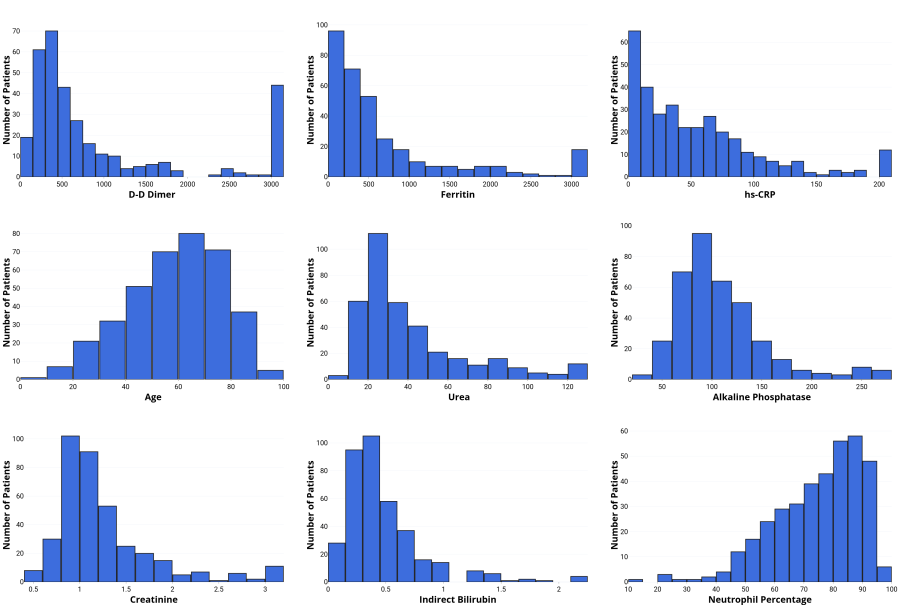


**Fig B:** Distribution of features with high predictive power.

**4. Reduced models**

## **4.1 Reduced Mortality prediction model:**

Features used (in order of decreasing SHAP feature importance): Age, neutrophils(%), creatinine, Urea, Alkaline phosphatase, serum sodium, indirect bilirubin, N/L ratio, Mean Corpuscular Hemoglobin, and AST/SGOT

| Training Dataset | 302 patients (165 alive + 137 dead) |
| --- | --- |
| Validation Dataset | 73 patients (40 alive + 33 dead) |
| Training accuracy | 88.74% |
| Validation accuracy | 84.93% |
| F-score | 0.83 |
| Sensitivity (Recall) | 0.85 |
| Specificity | 0.85 |
| PPV (Precision) | 0.82 |
| NPV | 0.85 |
| AUC-ROC (on validation set) | 0.91 |

**Table C:** Evaluation of reduced mortality model

##

## **4.2 Reduced Severity prediction model:**

Features used (in decreasing order of SHAP feature importance):

Age, Urea, High sensitivity C-reactive protein, D-D dimer, indirect bilirubin, AST / SGOT, monocytes(%), Red blood cell count, White blood cell count, ferritin

By reducing the number of features taken by the severity model and performing hyperparameter-tuning on the new model, we were able to achieve the following metrics:

| Training Dataset | 264 patients (146 severe + 118 non-severe) |
| --- | --- |
| Validation Dataset | 67 patients (37 severe + 30 non-severe) |
| Training accuracy | 87.12% |
| Validation accuracy | 86.57% |
| F-score | 0.88 |
| Sensitivity (Recall) | 0.89 |
| Specificity | 0.83 |
| PPV (Precision) | 0.87 |
| NPV | 0.83 |
| AUC-ROC (on validation set) | 0.93 |

**Table D:** Evaluation of reduced severity model

**References for Supplementary material**

1. Yeo I-K. A new family of power transformations to improve normality or symmetry [Internet]. Vol. 87, Biometrika. 2000. p. 954–9. Available from: <http://dx.doi.org/10.1093/biomet/87.4.954>

2. Huang, Z.: Clustering large data sets with mixed numeric and categorical values, Proceedings of the First Pacific Asia Knowledge Discovery and Data Mining Conference, Singapore, pp. 21-34, 1997.

3. McInnes L, Healy J, Melville J. UMAP: Uniform Manifold Approximation and Projection for Dimension Reduction [Internet]. 2018 [cited 2021 Aug 16]. Available from: <http://arxiv.org/abs/1802.03426>

4. Inc. PT. Collaborative data science [Internet]. Montreal, QC: Plotly Technologies Inc.; 2015. Available from: <https://plot.ly>
